# Prevalence of dementia in East Asia: a synthetic review of time trends

**DOI:** 10.1002/gps.4297

**Published:** 2015-05-11

**Authors:** Yu-Tzu Wu, Carol Brayne, Fiona E Matthews

**Affiliations:** 1 Department of Public Health and Primary Care, Cambridge Institute of Public Health, Forvie Site, University of Cambridge, School of Clinical MedicineCambridge, UK; 2 MRC Biostatistics Unit, Cambridge Institute of Public Health, Forvie Site, University of Cambridge, School of Clinical MedicineCambridge, UK

**Keywords:** dementia, epidemiology, review of the literature, East Asia, old age

## Abstract

**Objective:**

This study aims to synthesise evidence on time trends of dementia prevalence in East Asian countries including Japan, China, South Korea and Taiwan and assess the impact of the societal changes on future prevalence.

**Method:**

Relevant reviews and recent nationwide studies in East Asia were identified to investigate changes in prevalence of dementia over time taking into account the potential impact of methodological factors and study designs.

**Results:**

The robust evidence that has been interpreted to suggest a substantial increasing trend over time is less compelling once fundamental differences in study methods and populations across individual surveys are considered. In Japan, longitudinal studies in small areas suggest the potential increase of prevalence after 2000. Increasing trends in China, South Korea and Taiwan over the last 20–30 years are based on the literature review without adjustment for methodological differences. Economic development and huge societal changes alongside the rise of non-communicable disease in East Asia could lead to increasing prevalence of dementia in the future once those cohorts with high risk of dementia reached their older age.

**Conclusion:**

Current evidence is not sufficient to suggest increasing trends of dementia prevalence in East Asia. Longitudinal studies with representative samples and stable methodology are needed to provide fundamental information of the epidemiology of dementia and identify important risk factors in East Asian societies. © 2015 The Authors. *International Journal of Geriatric Psychiatry* published by John Wiley & Sons Ltd.

## Introduction

Population ageing has been a global public health issue (United Nations, 2013). Especially in East Asia, extended life expectancy and considerable number of older people are expected to have substantial impact not only on health care systems but also on the whole societies (Aggarwal *et al*., 2012; Catindig *et al*., 2012). Governments in East Asian countries have experienced increasing burden of non-communicable diseases and mental illness with a particular concern about dementia, a syndrome of cognitive decline with loss of basic functioning for daily life. Based on the report from the World Health Organisation in 2012, it is estimated that of the 35 million of people with dementia, nearly a quarter live in East Asia and the number is expected to double every 20 years (World Health Organisation, 2012). Dementia prevalence trends over time, and estimations for the near future are of interest to both research communities and worldwide governments for policy planning.

The prevalence of dementia in Europe and the USA is considered to be stable or reduced over the last decades (Larson *et al*., 2013). Recent longitudinal studies with consistent study designs and methods provide robust evidence on comparing dementia prevalence over time periods with cohorts in high-income countries (Lobo *et al*., 2007; Matthews *et al*., 2013; Qiu *et al*., 2013). In contrast, several reviews have suggested an increasing trend of prevalence over time in East Asian countries (Dodge *et al*., 2012; Yu *et al*., 2012; Alzheimer's Disease International's, 2013; Chan *et al*., 2013). However, these conclusions were mainly based on the summary of published studies. Without examining variation in methodological factors and changes in population structure very carefully, the integrated results of the individual prevalence studies from East Asia could be over-interpreted.

Dramatic transitions in East Asian countries over the last hundred years have improved the basic living conditions and health of population and might moderate the risk factors for dementia and the identification of clinical case over time. As well as synthesising the evidence in current literature, it is important to explore future trends in dementia prevalence from a holistic perspective, with the consideration of changes in social environment and population health profile. This review aims to provide a nuanced understanding of trends in dementia prevalence in East Asia. Instead of conducting a systematic review, this study is designed to be an in-depth review scrutinising the findings from different epidemiological studies. This study first presents the synthesised evidence on time trends of dementia prevalence in East Asian countries (Japan, China, South Korea and Taiwan) over the last few decades taking methodological variation into account and further considers the relationship between societal changes and future prevalence.

## Methods

A literature search was conducted in PubMed to identify population-based studies and reviews related to trends of dementia prevalence in East Asian countries including Japan, China, Korea and Taiwan until March 2015. The search terms included ‘dementia’ or ‘Alzheimer’, ‘prevalence’ or ‘epidemiology’ and ‘trend’ or ‘cohort’ or ‘review’. Instead of conducting an exhaustive review of existing surveys, this study particularly focused on three types of literature: (1) reviews of dementia prevalence in East Asia; (2) population-based studies comparing prevalence across time periods with consistent study designs and methodologies; and (3) recent nationwide studies (in the last 5 years). Existing reviews provide basic information on the epidemiological studies of dementia over the last decades. The true changes in dementia risk can only be detected by the second type of literature, which controlled for methodological difference. Because this type of studies was insufficient in East Asia, recent nationwide population-based studies were consulted to compare with the estimates in previous studies with the consideration of methodological variation. The results in the literature were synthesised by countries.

## Results: a synthesis of the evidence in East Asian countries

The literature search identified 648 papers, including eight reviews and two population-based studies comparing prevalence across time periods. Based on the reference lists of these papers, another two reviews and one study were further added. In total, this review included ten reviews, three prevalence trend studies and four nationwide studies in East Asian countries (Table 1).

**Table 1:** Reviews and studies related to trends in dementia prevalence

Reviews							
Author	Country/region	Year range of the review	Number of included studies	Databases	Meta-analysis	Adjusting for methodological factors	Main findings on prevalence trend
Dong *et al.*, 2007	China	1980–2004	25	English, Chinese	Yes	No information	Increased from 2.1% to 4.0% in people aged 60 years and over
Chan *et al*., 2013	China	1990–2010	76	English, Chinese	Yes	No but mentioned in the discussion	Substantial increase by 5-year age groups over the last two decades
Yu *et al*., 2012	Hong Kong	1995, 2005–2006	2	English	No	No	Increased from 4.5% in 1995 to 9.3% in 2005 based on two studies
Zhang *et al*., 2012	China, Hong Kong, Taiwan	1980-2010	73	English, Chinese	Yes	No information	Increased from 1.3% to 3.6% in people aged 55 years and over
Wu *et al*., 2014	China, Hong Kong, Taiwan	1980–2012	76	English, Chinese	Yes	Yes	No significant variation across time periods after adjusting for age groups and diagnostic criteria
Fuh and Wang, 2008	Taiwan	1980–1993	5	English, Chinese	No	No	Increased from 1.7% to 4.3% based on descriptive information from five studies
Dodge *et al*., 2012	Japan	1985–2008	5	English	No	No but explored the variation across studies	Increased by 10-year age group based on descriptive information
Okamura *et al*., 2013	Japan	1985–2012	21	English, Japanese	No	No	Increased from 2.9 to 12.5% based on descriptive information
Cho *et al*., 2011	South Korea	1994–2009	7	English	No	No	No clear trend with a range between 6.8% and 13.0%
Kim *et al.*, 2014	South Korea	1990–2013	11	English, Korean	Yes	No	No clear trend with a range between 7.3% and 10.1%
**Prevalence trend study**							
**Author**	**Country**/**region**	**Year of investigation**	**Number of investigation**	**Age range**	**Consistent diagnostic criteria**	**Response rate**	**Main findings**
Li *et al*., 2007	Beijing, China	1987/1997	2	60+ years	No (DSM-III-R/ICD-10)	81.9%, 93.5%	Increasing trend of prevalence from 1.7% to 2.5%
Sekita *et al*., 2010	Hisayama, Japan	1985/1992/1998/2005	4	65+ years	Yes (DSM-III-R)	Over 90% across four cohorts	Increasing trend of prevalence from 6.7%, 5.7%, 7.1% to 12.5%
Wakutani *et al*., 2007	Daisen Cho, Japan	1980/1990/2000	3	65 years+	Yes (DSM-III)	Unknown in 1980/1990; 85% in 2000	Increasing trend of prevalence from 4.4%, 4.9% to 7.4%
**Recent nationwide study**							
**Author**	**Country**/**region**	**Year of investigation**	**Sample size**	**Age range**	**Diagnostic criteria**	**Response rate**	**Crude prevalence**
Jia *et al*., 2014	China	2008–2009	10276	65+ years	DSM-IV	74.4%	5.1% (95% CI: 4.7, 5.6)
Sun *et al*., 2014	Taiwan	2011–2013	10432	65+ years	NIA-AA	36.5%	6.3% (95% CI: 5.6, 7.0)
Ikejima *et al*., 2012	Japan	2009	3394	65+ years	DSM-III	54.7%	22.5% (95% CI: 21.2, 24.1)
Kim *et al.*, 2014	South Korea	2008	6141	65+ years	DSM-IV	71.6%	9.2% (95% CI: 7.9, 10.4)

DSM-IV, The Diagnostic and Statistical Manual of Mental Disorders, Fourth Edition; DSM-III, Diagnostic and Statistical Manual of Mental Disorders, Third Edition; DSM-III-R, Diagnostic and Statistical Manual of Mental Disorders, Third Edition, Revised; ICD-10, the International Classification of Diseases 10th edition; NIA-AA, National Institute on Aging-Alzheimer's Association criteria; CI, confidence interval.

### China

Dementia prevalence in mainland China has been reported to be rising in recent reviews (Dong *et al*., 2007; Zhang *et al*., 2012; Chan *et al*., 2013). From 1990 to 2010, estimates of age-specific prevalence doubled or nearly tripled across age groups (Chan *et al*., 2013). However, this increasing trend could be a function of changes in study designs and methodological factors such as diagnostic criteria, which have been revised considerably in the past 30 years. A systematic review taking these into account did not show significant changes, and that there is major heterogeneity between studies (Wu *et al*., 2014). Prevalence estimates in the studies using newer diagnostic criteria such as the Diagnostic and Statistical Manual of Mental Disorders, Fourth Edition (DSM-IV) and 10/66 diagnostic algorithm are systematically and significantly higher than those using older diagnostic criteria such as the Diagnostic and Statistical Manual of Mental Disorders, Third Edition (DSM-III), Diagnostic and Statistical Manual of Mental Disorders, Third Edition, Revised (DSM-III-R), Chinese Classification of Mental Disorders (Wu *et al*., 2013). Temporal variation of prevalence became less clear after adjusting for diagnostic criteria and age structure. One study compared the prevalence in a well-defined population in Beijing over 10 years (1986 and 1997) (Li *et al*., 2007). Although the prevalence was reported to increase over time in the same area, the diagnostic criteria were not consistent over the two time periods. Similar issues related to varying study methods were also found in another review, which reported an increasing prevalence in Hong Kong over 10 years (Yu *et al*., 2012). A recent multicentre study applying DSM-IV criteria in urban and rural areas of mainland China reported stable estimated prevalence (World Health Organisation, 2012; Jia *et al*., 2014). The increase of dementia prevalence in China has probably been exaggerated because of changes in methodological factors over the period of the reported studies.

### Japan

Recent reviews have also reported an increasing trend of dementia prevalence in Japan since 1980s (Dodge *et al*., 2012; Okamura *et al*., 2013). However, variation of study methods (such as diagnostic criteria of the International Classification of Diseases 9th edition and 10th edition, DSM-III, DSM-III-R, DSM-IV and Diagnostic and Statistical Manual of Mental Disorders, Fourth Edition, Revised) and characteristics of study population (such as whole study age range of 60+ or 65+ years) over time have not been fully controlled. Similar to China, more recent studies using newer diagnostic criteria have been more likely to report higher prevalence (Dodge *et al*., 2012; Wada-Isoe *et al*., 2012). Based on the estimates in the community-based studies using the similar study methods and diagnostic criteria in the same geographical areas, stable dementia prevalence was reported from 1980s to 1990s, but then a particularly high prevalence was reported in the surveys after 2000 (Wakutani *et al*., 2007; Sekita *et al*., 2010). Although these studies were conducted in small areas with potential concerns regarding study designs and analytical methods, the difference of adjusted prevalence between pre and post-2000 periods was about 3%. A recent nationwide study using the traditional diagnostic criteria also reported a high prevalence in the population aged 65 years and over (Ikejima *et al*., 2012). The prevalence of dementia in Japan appeared to have been relatively stable before the year 2000 with potential increases in more recent years.

### South Korea

The prevalence of dementia in South Korea is generally high in the range of 6–10% from the studies in mid-1990s (Cho *et al*., 2011; Kim *et al.*, 2014). Although full adjustment was not carried out to reflect changes in demographic factors, the estimates using different diagnostic criteria (DSM-III-R, DSM-IV and 10/66 diagnostic algorithm) were not substantially different. Comparing new nationwide surveys in Korea with other countries, the prevalence by 5-year age groups in the Korean study was nearly double the recent European and Chinese studies (Lobo *et al*., 2007; Kim *et al*., 2011; Qiu *et al*., 2013; Jia *et al*., 2014). Limited pre-1990 data and lack of studies that aim to compare the prevalence across time periods cause difficulty in explaining temporal variation of dementia prevalence in Korea.

### Taiwan

Most of the prevalence studies in Taiwan were conducted in 1990s but across different geographical areas (Fuh and Wang, 2008). Although similar diagnostic criteria have been used, these surveys had varying study designs and population sampling methods and might not provide sufficient information to detect temporal variation in the community-based population over recent decades. A recent nationwide study reported higher prevalence than the previous studies (Sun *et al*., 2014). Although this study provides newer estimates of prevalence in Taiwan, the variation of diagnostic criteria (DSM-III, DSM-III-R and the National Institute on Aging-Alzheimer's Association criteria) and measurement methods could impede valid comparison.

## Discussion

### Increasing prevalence?

Although the existing studies and reviews look at face value as though there might be changing prevalence of dementia in East Asian countries, a more rigorous approach taking methods and populations into account suggests this might be premature. In Japan, longitudinal studies in small areas do support a potential increase of prevalence after 2000. In China, South Korea and Taiwan, the apparent increase over the last 20–30 years was not so clear and needs to be explored with more comparable data across time. Prevalence in South Korea is higher than other East Asian countries in a stable manner without obvious temporal variations.

### Variation and factors beyond methodology

Although some studies have retained similar diagnostic criteria and measurement methods over time, residual variation ‘beyond methodological factors’ is quite possible. Training of clinicians, knowledge and attitudes to dementia in professionals has also changed over decades and affect diagnostic standards and measurement methods. Even though researchers might attempt to ensure stability over time, it is difficult to avoid the influence of different applications between the clinicians and the pervasive influence of changing attitude to diagnostic boundaries.

The stigma of mental illness and dementia reported in the earlier prevalence studies might have led to difficulty and resistance in identifying people with dementia and other mental disorders in older age (Kleiman, 1996; Wu *et al*., 2013; Chiu *et al*., 2014). Cognitive decline in later life has been interpreted as a natural stage of ageing in many societies rather than a common mental disorder. In recent years, awareness campaigns, conducted by charitable organisations for people with dementia and their carers, have had a major impact in bringing knowledge of dementia to the general public with increasing governmental involvement. Societies in East Asian countries now have a more positive attitude about discussions related to dementia and cognitive decline in later life (Chiu *et al*., 2014). People with dementia are now more likely to seek help from medical and health care professionals rather than resolve within their family. As in many countries, including continental Europe, dementia in East Asia is mainly considered to be a neurological dysfunction rather than a psychiatric syndrome and usually treated, diagnosed and studied by neurologist. All these factors could play a part in apparent changes over time.

The rise of political interest on dementia in recent years is having a fundamental impact on dementia research. Ageing populations in East Asia are a potential huge market and will be focus of the interest of medical industries, insurance companies and health and social care business. Taking the apparent increasing trends provides strong support for those calling for more investment and research funding from both public and private sectors, particularly for early detection. Such calls might influence research environment and substantially moderate the results and interpretations of epidemiological studies.

### Societal changes, changing risk factors and dementia

The literature on epidemiological transitions has focused on the complex change in patterns of health and diseases and their interactions with demographic, economic and sociologic determinants (Omran, 2005). Three stages of transition (the Age of Pestilence and Famine, the Age of Receding Pandemics and the Age of Degenerative and Man-Made Diseases) have been proposed and supported by three types of transition model (Classical, Accelerated and Delayed). Dementia, which should be included in the third age (the Age of Degenerative and Man-Made Diseases), is highly related to age, changing life expectancy and health profiles at population levels alongside societal level factors.

**Figure 1:**
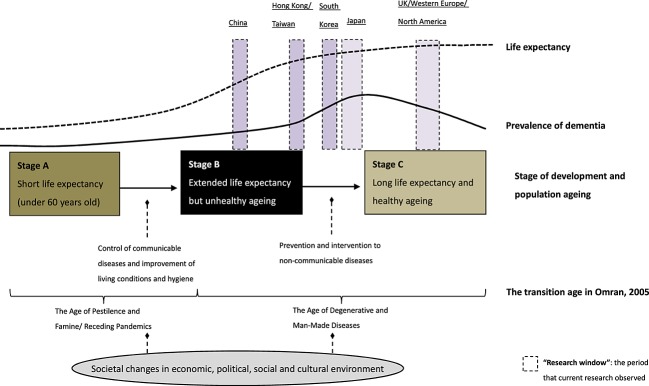
Societal changes, extended life expectancy and time trends in prevalence of dementia.

In East Asia, dramatic societal changes and increased life expectancy over the last decades would be expected to influence the risk for each successive cohort and indeed change dementia prevalence in the near future. It is possible to hypothesise a relationship between societal changes, life expectancy and the prevalence of dementia (Figure1). The ‘research window’ shows the period of available data for prevalence, which covers 20–30 years from Japan, along with Western European and North American countries, starting investigation of the epidemiology of dementia in the 1980s while low and middle income countries such as China, Korea and Taiwan started over a decade later.

The prevalence of dementia in Figure1 is assumed to be age-standardised, that is, taking changes in age structure into account. Although increasing prevalence of dementia over time can be substantially attributed to population ageing, extended life expectancy, an indicator for the health of populations, is also likely to contribute additional influence on dementia occurrence.

#### Stage A: low life expectancy and ‘healthy survivor’

In the first stage of development and population ageing, a country usually had low life expectancy, and only a small proportion of people survived over 60 years old (Stage A). Because most people died younger and often related to poor living conditions such as famine, wars and infectious diseases, it could be argued that those who survived to their older age were more resilient with better health status and lower risk of cognitive decline and dementia at older age. Because of this ‘healthy survivor’ effect, the prevalence of dementia might be low at this stage of societal development even taking age structure into account. The lower prevalence estimates in African countries (age-standardised) in the global prevalence review may support this interpretation of this stage (Prince *et al*., 2013).

Life expectancy in East Asia has lengthened considerably over the last decades. All these countries had the life expectancy at birth below 40 years old at the beginning of the 20th century reaching from 75 (China) to 85 (Japan) years in 2014 (Central Intelligence Agency, 2014). Life expectancy in Japan has generally been higher than other countries and achieved 70 years in mid 1960s. During the same time period, life expectancy in China and South Korea was less than 60 years with dramatic fluctuations. In Taiwan and Hong Kong, which have different colonial histories from China, it has been closed to the estimates from Japan.

#### Stage B: extended life expectancy but unhealthy ageing

With economic development, improved living conditions and prolonged life expectancy, an increasing number of people now survive to their older age (Stage B). Rapid reduction in competitive cause of death such as tuberculosis, pneumonia, diarrhoea, starvation and injury means a large number of people are surviving to older ages. Over the second half of the 20th century, East Asian countries have addressed the issues of famine, wars and communicable diseases and achieved high life expectancy in a relatively short period compared with Western countries, other low and middle income countries. Although the child mortality has been decreased considerably because of improved living conditions, these generations, generally born before 1950, might have experienced dramatic changes during their younger ages with poor nutrition, none or low education. In their adulthoods, unhealthy lifestyle (smoking, alcohol abuse and high-demanding occupation) and the threat of non-communicable diseases (diabetes, vascular diseases and hypertension) are recognised as major health threats to these cohorts. This is supported by recent studies reporting increasing prevalence of obesity, metabolic syndrome and other non-communicable diseases and their potential associations with changes in lifestyle factors such as westernised eating patterns (Lao *et al*., 2014; Mukai *et al*., 2014; OECD, 2014). Several of these are known risk factors for dementia and cognitive decline (Norton *et al*., 2014). Risk factors at earlier life stages may have substantial impact on cognition in later life and result in a time–lag relationship between the extended life expectancy and the increasing trend of dementia prevalence.

Thus in these countries, cohorts with adverse early life experiences are surviving, potentially with increased risk of dementia occurrence. The population-based studies in Japan, the first developed country in East Asia, have reported a potential increase of dementia prevalence over time (Wakutani *et al*., 2007; Sekita *et al*., 2010). Considering the rise of non-communicable diseases in East Asian countries, the prevalence of dementia could indeed increase in the next few decades. This includes China, the country with the largest population in the world, even though the trend of prevalence has not significantly increased over the last 30 years (Wu *et al*., 2014).

#### Stage C: extended life expectancy and healthy ageing

High-income countries in Western Europe and North America can be considered to be in Stage C. Compared with Stage B, people in high-income countries have not only long life expectancy but also stable social environment and living conditions over the last decades. Their early lives were characterised by relatively positive health and risk profile. Based on recent studies, a stability or reduced prevalence of dementia in European populations might to be attributed to high education attainment, modification of lifestyle factors and non-communicable diseases (Larson *et al*., 2013; Matthews *et al*., 2013). Interventions for lifestyle factors and primary prevention of non-communicable diseases at population levels could substantially reduce not only the risk of vascular diseases and metabolic syndromes in middle age but also the potentially dementia occurrence in later life. This could be related to the hypothesis of compression of morbidity, which suggests that chronic conditions can be postponed and compressed into the shorter span before death by changes in lifestyle (Fries, 1980). In addition to individual lifestyle and health behaviour, findings from East Asian countries might further suggest the impact of dramatic societal changes on population health in later life.

**Figure 2:**
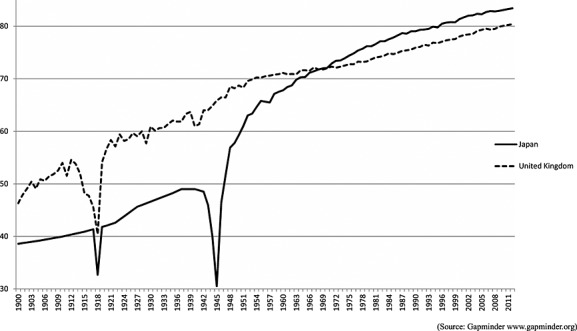
Life expectancy at birth in the UK and Japan.

Although Japan is included in the high-income group as Western Europe or North American countries, the older people included in the research window may have lived in very different conditions particularly in the first half of the 20th century. After the World War II, Japanese society, as well as other East Asian countries, experienced substantial transitions of social environment with earlier economic development and dramatic increase of life expectancy in the 1960s. Previous literature has described the transition pattern of Japan using the Accelerated model, which has much faster shift to the Age of Degenerative and Man-Made Diseases compared with the Classical model in Western countries (Omran, 2005). In the UK, life expectancy at birth approached 60 years in the 1920s, but Japan only achieved this in the early 1950s with the adverse impact of the Second World War (Figure2). Although Japan has higher life expectancy than UK now, the differences in their histories might result in different trends in dementia prevalence over time. Lifecourse experience of Japanese cohorts will have been very different from the western society.

### Public health implications and future research directions

Although current evidence does not prove increasing trends in dementia prevalence in East Asia, the prevalence of dementia may well increase in the future given the rise of obesity and non-communicable diseases. Instead of only focusing on medical service and health care on individual level, policy planning in East Asian countries needs to address the substantial determinants of poor health outcomes across the lifecourse and create a healthy environment for whole societies including their ageing populations.

Although the healthcare databases can provide information of medical service usage over time, health conditions in general populations need to be investigated through primary data collection. In East Asia, rapid societal development and the accelerated transition of epidemiology are unique, different from the graduate changes in western societies. Repeated longitudinal studies with representative samples and stable methodologies will be required to provide this fundamental information of the epidemiology of dementia in community-based populations and identify important risk factors in East Asian societies. Because the number of people with dementia and rapid population ageing remains important concerns to health, social and economic systems in the near future, epidemiological studies are essential for an evidence-based approach to assist in developing public health policy in East Asia.

## Conflict of interest

None declared.

**Key points**

- This synthetic review summarises evidence on time trends of dementia prevalence in East Asian countries (Japan, China, South Korea and Taiwan) and assesses the impact of the societal changes on future prevalence.
- Although current evidence is not sufficient to suggest increasing trends of dementia prevalence in East Asia over the last few decades, economic development and huge societal changes alongside the rise of non-communicable disease could lead to increasing prevalence of dementia in the future once those cohorts with high risk of dementia reached their older age.
